# Camptothecin Regulates Microglia Polarization and Exerts Neuroprotective Effects *via* Activating AKT/Nrf2/HO-1 and Inhibiting NF-κB Pathways *In Vivo* and *In Vitro*

**DOI:** 10.3389/fimmu.2021.619761

**Published:** 2021-04-01

**Authors:** Dewei He, Shoupeng Fu, Ang Zhou, Yingchun Su, Xiyu Gao, Yufei Zhang, Bingxu Huang, Jian Du, Dianfeng Liu

**Affiliations:** College of Animal Science and Veterinary Medicine, Jilin University, Changchun, China

**Keywords:** camptothecin, Parkinson’s disease, M1/M2, microglia polarization, neuroinflammation

## Abstract

Microglia, the main immune cells in the brain, participate in the innate immune response in the central nervous system (CNS). Studies have shown that microglia can be polarized into pro-inflammatory M1 and anti-inflammatory M2 phenotypes. Accumulated evidence suggests that over-activated M1 microglia release pro-inflammatory mediators that damage neurons and lead to Parkinson’s disease (PD). In contrast, M2 microglia release neuroprotective factors and exert the effects of neuroprotection. Camptothecin (CPT), an extract of the plant *Camptotheca acuminate*, has been reported to have anti-inflammation and antitumor effects. However, the effect of CPT on microglia polarization and microglia-mediated inflammation responses has not been reported. In our study we found that CPT improved motor performance of mice and reduced the loss of neurons in the substantia nigra (SN) of the midbrain in LPS-injected mice. In the mechanism study, we found that CPT inhibited M1 polarization of microglia and promotes M2 polarization *via* the AKT/Nrf2/HO-1 and NF-κB signals. Furthermore, CPT protected the neuroblastoma cell line SH-SY5Y and dopaminergic neuron cell line MN9D from damage mediated by microglia activation. In conclusion, our results demonstrate that CPT regulates the microglia polarization phenotype *via* activating AKT/Nrf2/HO-1 and inhibiting NF-κB pathways, inhibits neuro-inflammatory responses, and exerts neuroprotective effects *in vivo* and *in vitro*.

## Introduction

Parkinson’s disease (PD), the most common neurodegenerative disease, seriously affects human health. PD is characterized by the loss of dopaminergic neurons in the substantia nigra (SN) of the midbrain, and its clinical manifestations are motor dysfunction. At present, it is generally believed that environmental, genetic, physiological aging, and other factors can cause PD (1, 2). The specific pathogenesis is not yet clear. Accumulated evidence has shown that neuroinflammation participated in the occurrence and development of PD (3, 4). Neuroinflammation has a two-way regulation effect. On the one hand, when the central nervous system is damaged by various pathogenic factors, immune cells are activated, and the resulting inflammatory response can kill harmful pathogens, remove abnormally accumulated proteins and cell fragments, and maintain damaged neurons, playing a role in protecting neurons. On the other hand, when the inflammatory reaction continues, various harmful factors will be released and accumulate in the brain, such as inflammatory chemokines, reactive oxygen species, excitatory amino acid ions, etc., which cause damage to peripheral neurons and lead to PD being further exacerbated (5–7). Therefore, proper regulation of neuroinflammation is of great significance for alleviating PD.

Microglia, a type of immune cells that exist in the central nervous system (CNS), have the functions of immune monitoring, phagocytosis of debris, secretion of various factors, and antigen presentation (8). Microglia have three phenotypes: M0, M1, and M2. Under normal conditions, microglia are in the M0 phenotype. Activated microglia are classified as M1-like (showing pro-inflammatory signals and neurotoxicity) or M2-like (involved in remissions of inflammation) based on the presence of specific cell surface molecules and expression of specific cytokines. M1, the pro-inflammatory phenotype, produces pro-inflammatory mediators and chemokines to promote tissue defense and pathogen destruction. However, persistent M1 microglia continue to participate in the pathogenesis of PD and amplify neuronal damage caused by pathological stimuli and toxins, which in turn lead to more extensive damage to neighboring neurons. M2, replacement macrophages, can release CD206, Arg-1, Ym-1, and other molecules to promote wound healing and tissue repair. Arg-1 is involved in arginine metabolism. On the one hand, it competes with the arginine of iNOS and reduces the release of NO. On the other hand, enzyme products of the Arg-1 pathway play an important role in tissue repair. Its products, polyamines (such as spermine), are polyvalent cations needed for cell proliferation and differentiation and help protect neurons from damage using pro-inflammatory cytokines. CD206 is a characteristic of selective activation in spinal cord injury, which promotes central nervous system repair while inhibiting secondary inflammation-mediated injury. Ym-1 is a 45 kDa secreted protein synthesized by activated peripheral macrophages. It binds to sugars and heparin sulfate on the cell surface to help protect the ECM scaffold at the injured site and play a neuroprotective role (9–11). Inhibiting pro-inflammatory microglia and promoting anti-inflammatory microglia are of great significance for inhibiting neuroinflammation and alleviating PD.

Camptothecin (CPT), a plant drug extracted from *Camptotheca acuminate*, has rich pharmacological effects such as being antivirus, antitumor, and causing changes in the keratinization process of the skin epidermis (12, 13). CPT has a wide range of sources and is easy to extract. Most previous studies have focused on the therapeutic effects of CPT on cancer. High concentrations of CPT can inhibit the malignant proliferation of cells and exert anti-tumor effects (14). However, high doses of CPT also affect normal cells and show side effects (15, 16). Therefore, the clinical application of CPT is limited. Studies have found that low concentrations of CPT can show an anti-inflammation effect. In *in vivo* sepsis models, CPT can inhibit the expression of several inflammatory cytokines and rescue mortality caused by inflammation (17, 18). Studies showed that CPT at low concentrations can inhibit the expression of topoisomerase 1, which in turn has been found to inhibit the expression of inflammatory genes (19, 20). It is known that NF-κB is closely related to the polarization of macrophages. Studies have also reported that CPT affects the activity of NF-κB (21, 22). However, the effect of CPT on microglia polarization and microglia-mediated inflammation responses has not been reported. In our study, we aim to explore the effect of CPT on microglia polarization and its underlying mechanism on neuroinflammation.

## Materials and Methods

### Reagents and Chemicals

Camptothecin (CPT, purity ≥98%) was bought from Pu Feide Biotechnology (Chengdu, China). Lipopolysaccharide (LPS, E. coli, Serotype O55:B5) and Dulbecco’s modified Eagle’s medium (DMEM) were obtained from BestBio (Shanghai, China). Fetal bovine serum (FBS) and horse serum (HS) were bought from Gibco (Grand Island, USA). TRIzol reagent was provided by Sigma-Aldrich (StLouis, U SA). The 0.05% trypsin was bought from Invitrogen (Carlsbad, USA).

### Animals and Models

Sixty male C57BL/6 mice (25-30 g) were purchased from Liaoning Changsheng Technology (Liaoning, China). Our experiment was recognized by the Institutional Animal Care and Use Committee of Jilin University (Changchun, China) (Permit Number: 2015047). During the experiment, we did our best to reduce the suffering of animals. The PD mouse model was established. The experiment included four groups: control group (which was injected PBS into the right SN), treated with CPT (1 mg/kg) group, LPS-injected group, and LPS-injected treated with CPT (1mg/kg) group. The mice were injected with LPS (5 µg/µl, 2 μL) or equal volumes of PBS into the right SN after anesthesia (stereotactic coordinates: dorsoventral (DV) = – 2.5 mm, mediolateral (ML) = – 0.8 mm and anterior-posterior (AP) = + 0.5 mm.). CPT was dissolved in PBS and given by gavage every two days. In addition, mice received CPT 3 days before surgery and for a total of 24 days. Before the operation and on the 28th day after the operation, we measured the body weight of the mice. On the 24th day after the operation, we performed an open field test.

### Open Field Test

On the 24th day after LPS injection, the open field assay was performed to test the effect of the CPT on the motor activity of mice. Mice were placed in a quiet, low-light box and allowed to adapt to the environment for 5 min. The bottom area of the box was cleaned up prior to the test to avoid the effect of previous tests. The experimental mice were placed in the open field for 5 min. The trajectory, total traveled distance, and time in the center square within 5 min were recorded by the computer and software (Any-maze, Stoelting Co).

### Immunohistochemistry

After being stripped, the brains of mice were fixed in 4% formaldehyde and paraffin sectioned as described previously (23). The immune-histochemical processes were conducted using a Ultra-Sensitive S-P kit. The dopaminergic neurons and microglia in SNpc were marked using the anti-tyrosine hydroxylase (TH) (1:800) (Abcam, Cambridge, UK) and anti-ionized calcium-binding adaptor molecule-1 (IBA-1) (1:1000) (Abcam, Cambridge, UK). The results were viewed under a microscope and the TH- and IBA-1-positive cells were analyzed by Image J-cell counter.

### Cell Culture

Microglia cell line BV-2, the neuroblastoma SH-SY5Y, and dopaminergic neuron MN9D cell were obtained from the Cell Culture Center at the Institute of Basic Medical Sciences (Peking, China). The cells were cultured in DMEM medium which contained 10% FBS (Gibco, Grand Island, NY, USA). The medium was changed every day and the cells were passaged when the confluence of the cells reached 70-80%. In our study, cells were treated with CPT for 1 h, stimulated with LPS (1μg/mL) for different times, and then collected for measurement.

### Reverse Transcription Quantitative Real-Time Polymerase Chain Reaction (RT-PCR)

Total RNA of the cells and tissues were collected with the RNA extraction kit (Takara Biotechnology, Ltd., Kyoto, Japan). After detection of RNA concentration, 3 μg RNA was reverse-transcribed into cDNA with the PrimerScript^®^ 1^st^ Strand cDNA Synthesis Kit (Sigma-Aldrich, St. Louis, MO, USA). After that, the mRNA levels of M1 markers (IL-6, TNF-α, COX-2 and iNOS) and M2 markers (CD206, Ym-1 and Arg-1) were examined with the SYBR Green Kit (South San Francisco, CA, USA) and the results were analyzed relative to β-actin using the 2^-ΔΔCt^ means. The sequences of the factors were shown in **Table 1**, which referred to previous studies (24).

**Table 1:** The primer sequences for RT-PCR.

Gene	sequences
*IL-6*	(F) 5’- CCAGAAACCGCTATGAAGTTCC-3’ (R) 5’- GTTGGGAGTGGTATCCTCTGTGA-3’
*TNF-α*	(F) 5’-GCAACTGCTGCACGAAATC-3’ (R) 5’-CTGCTTGTCCTCTGCCCAC-3’
*iNOS* *COX-2*	(F) 5’-GAACTGTAGCACAGCACAGGAAAT-3’ (R) 5’-CGTACCGGATGAGCTGTGAAT-3’ (F) 5’-CAGTTTATGTTGTCTGTCCAGAGTTTC-3’ (R) 5’-CCAGCACTTCACCCATCAGTT-3’
*Arg-1* *Ym-1* *CD206* *β-actin*	(F) 5’-GTGAAGAACCCACGGTCTGT-3’ (R) 5’-GCCAGAGATGCTTCCAACTG-3’ (F) 5’-CAGGGTAATGAGTGGGTTGG-3’ (R) 5’-CACGGCACCTCCTAAATTGT-3’ (F) 5’-CTTCGGGCCTTTGGAATAAT-3’ (R) 5’-TAGAAGAGCCCTTGGGTTGA-3’ (F) 5’-GTCAGGTCATCACTATCGGCAAT-3’ (R) 5’-AGAGGTCTTTACGGATGTCAACGT-3’

### Western Blot

Total proteins of the cells and tissues were obtained with a protein assay kit and then quantified into 40 μg per 15 μl using SDS and P0013B lysate (Beyotime Inst. Biotech, Beijing, China) for western blot assay. The nucleoprotein and plasmid protein were extracted according to the instructions of the nucleoprotein extraction kit (Beyotime Inst. Biotech, Beijing, China). A total of 40 μg of proteins were subjected to 8%, 12%, and 15% SDS-PAGE and transferred onto PVDF membranes (Millipore, Billerica, MA, USA). After being blocked with 5% skim milk, membranes were incubated for 12 h with primary antibodies against TH(1:500) (Abcam, Cambridge, UK), IBA-1 (1:500) (Abcam, Cambridge, UK), COX-2 (1:1000) (Abcam, Cambridge, UK), iNOS (1:1000) (Abcam, Cambridge, UK), phos-AMPK (1:1000) (Abcam, Cambridge, UK), AMPK (1:1000) (Abcam, Cambridge, UK), phos-AKT (1:1000) (Abcam, Cambridge, UK), AKT (1:3000) (Abcam, Cambridge, UK), phos-NF-κB p65 (1:3000) (Abcam, Cambridge, UK), NF-κB p65(1:3000) (Abcam, Cambridge, UK), Nrf2(1:1000) (Santa Cruz, CA, USA), HO-1(1:2000) (Santa Cruz, CA, USA), and β-actin (1:5,000) (Santa Cruz, CA, USA). Next, the membranes were washed six times with TBST (5 min per time) and incubated for 2 h against the secondary antibodies for goat anti-mouse (1:4000) (Beyotime Inst. Biotech, Beijing, China) or goat anti-rabbit (1:4000) (Beyotime Inst. Biotech, Beijing, China). Then membranes were again washed six times with TBST (5 min per time). After that, the proteins were measured with ECL Luminous liquid (Beyotime Inst. Biotech, Beijing, China).

### Cytotoxicity Assay

The CCK-8 reagent contained WST-8 [chemical name: 2 - (2-methoxy-4-nitrophenyl) - 3 - (4-nitrophenyl) - 5 - (2,4-disulfonic acid benzene) - 2H tetrazole monosodium salt], which was reduced to a highly water-soluble yellow formazan dye (formazan dye) by dehydrogenase in cells under the action of electron carrier 1-methoxy-5-methylphenazionium dimethyl sulfate (1-methoxy PMS). The quantity of the generated armor is directly proportional to the quantity of the living cells. Therefore, this characteristic can be used for cell proliferation and toxicity analysis directly. In the study, we measured the effects of CPT on cell viability using the CCK-8 method. After being cultured for 24 h, the cells were incubated with DMSO and different concentrations CPT (0–2μM) for 20 h, and then CCK-8 diluent was added to the culture medium and cultured for 3 h. After that, the absorbance was examined with a microplate reader at 450 nm.

### ELISA

The cells were cultured in the 24-well plates. After incubation with CPT for 1 h and being stimulated with LPS (1 μg/mL) for 24 h, the protein expression of TNF-α and IL-6 in the supernatant was examined with the ELISA kits (R&D Systems, Abingdon, UK).

### Immunofluorescence Staining

Immunofluorescence staining was performed to determine the nuclear translocation of the NF-kB p65 in LPS-exposed BV-2 cells. The well-grown BV2 cells were inoculated on the sliders; when the cell density was about 60%, we pretreated cells with CPT (1 h) and added LPS to stimulate cells (1h), then fixed cells using paraformaldehyde for 15 min and perforated with 0.1% Triton-X100 for 5 min. After that, we blocked cells using donkey serum for 4 h. The sliver was then incubated with a primary antibody against the NF-κB p65(1:100) (Bio Mol Research Laboratories, Inc., Plymouth, PA) and IBA-1(1:100)(Abcam, Cambridge, UK). Then we combined this with the donkey anti-rabbit antibody (1:1000) that contains fluorescence. Then, after the nucleus was labeled with DAPI, we observed the result using a fluorescence microscope

### Data Analyses

Results were presented with mean ± SD, and analyzed with SPSS 13.0 software package (SPSS Inc., Chicago, USA). The differences were evaluated with the one-way analysis of variance (ANOVA) method. p < 0.05 was considered to be significant and p < 0.01 was considered to be markedly significant.

## Results

### CPT Treatment Alleviates the Weight Loss and Behavioral Disorder of LPS-Injected PD Mice

**Figure 1:**
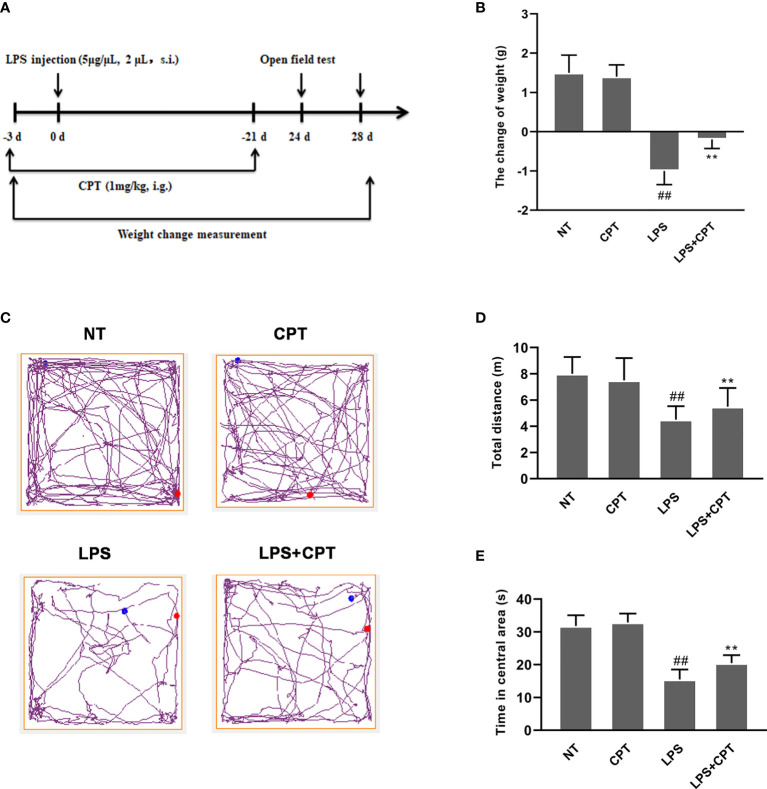
CPT treatment alleviates the weight loss and behavioral disorder of LPS-injected PD mouse model. **(A)** Experiment procedure. Mice were injected with LPS (5 μg/μL, 2μL) or equal volume PBS into the right SNpc after anesthesia and treated every two days with CPT (1 mg/kg) for 3 days before surgery and for a total of 24 days. **(B)** The weight change of mice during the experiment. Before the operation and on the 28th day after the operation, the body weight of mice was measured. **(C-E)** The locomotor activity of mice was tested using the open field assay. Results are shown as means ± SD (n= 5). *^##^p < 0.01* vs. the no-treatment (NT) group; **p < 0.01 vs. the LPS-injected group.

Unilateral injection of LPS into the SN can cause weight loss and motor dysfunction in mice. In order to prove the protective effect of CPT, we studied the effect of CPT treatment on weight loss and motor behavior disorders in the LPS-injected PD mouse model. The experimental process is executed as shown in **Figure 1A**. On the 28th day after LPS injection, the weight change of the mice was measured. The results showed that LPS injection caused weight loss in mice, and CPT treatment could significantly improve LPS-induced weight loss in the LPS-injected PD mouse model (**Figure 1B**). On the 24th day after LPS injection, we tested the athletic ability of mice through an open field experiment. The results showed that LPS injection caused the distance moving in the open field and time in the center to be less, and CPT treatment could improve this phenomenon (**Figures 1C–E**). These results illustrated that CPT treatment alleviates the weight loss and behavioral disorder of LPS-injected PD mouse model.

### CPT Treatment Decreases Dopaminergic Neurons Degeneration and Inhibits the Over-Activation of Microglia in LPS-Injected PD Mice

**Figure 2:**
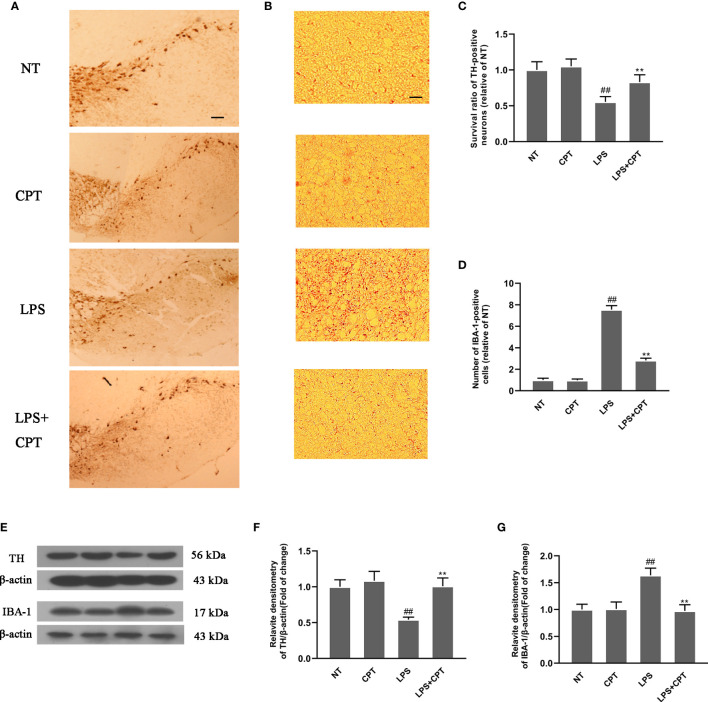
CPT treatment decreases dopaminergic neurons’ degeneration and over-activation of microglia in LPS- injected PD mice. Experiment mice were anesthetized and sacrificed to obtain the brains on the 28th day after LPS injection. **(A, C)** The numbers of TH-positive cells in the SN were examined using immunohistochemistry (the scale bar represents 200 μm). **(B, D)** The numbers of IBA-1-positive cells in the SN were examined using immunohistochemistry (the scale bar represents 50 μm). **(E–G)** The protein level of TH and IBA-1 in the midbrain was examined using western blot. Results are shown as means ± SD (n= 5). *^##^p < 0.01* vs. the no-treatment (NT) group; **p < 0.01 vs. the LPS-injected group.

The main pathological feature of PD is the degeneration of dopaminergic neurons and over-activation of microglia. To explore the protective effect of CPT, we studied the effect of CPT on the dopaminergic neurons and microglia in LPS- injected PD mice. On the 28th day after LPS injection, we obtained the brain tissues of mice and examined the number of TH and IBA-1-positive cells using the immunohistochemistry method. Results showed that LPS injection caused a significant decrease of dopaminergic neurons in the SN and over-activation of microglia in LPS- injected PD mice, and CPT treatment could protect the neurons from the damage caused by LPS and inhibit the over-activation of microglia (**Figures 2A–D**). The protein level of TH and IBA-1 was examined using western blot. The results also confirmed the protective effect of CPT from a protein perspective (**Figures 2E–G**). These results illustrated that CPT treatment decreases dopaminergic neurons degeneration and over-activation of microglia in LPS- injected PD mice.

### CPT Treatment Inhibits Inflammatory Response and Regulates Microglia Polarization in LPS- Injected PD Mice

**Figure 3:**
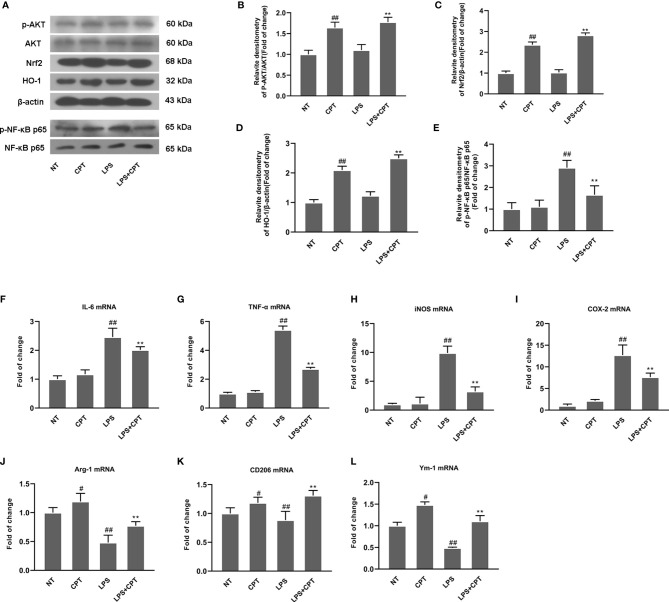
CPT treatment inhibits inflammatory response and regulates microglia polarization in LPS- injected PD mice. On the 28th day after modeling, the midbrain of mice was peeled off and ground. **(A–E)** AKT/Nrf2/HO-1 and NF-κB signal pathways were detected using western blot. **(F–L)** The mRNA levels of M1 markers (IL-6, COX-2, iNOS, and TNF-α) and M2 markers (Ym-1, CD206, and Arg-1) were detected using RT-PCR. Results are shown as means ± SD (n= 5).*^##^p < 0.01* vs. the no-treatment (NT) group; **p < 0.01 vs. the LPS-exposed group. ^##^p < 0.01 vs. the no-treatment (NT) group corrected to ^#^P < 0.05 and ^##^P < 0.01 vs. the no-treatment (NT) group.

To further confirm neuroprotection of CPT and its mechanism, we studied the effect of CPT on inflammatory response and microglia polarization in LPS- injected PD mouse model. On the 28th day after LPS injection, we obtained the brain tissues of mice. AKT/Nrf2/HO-1 and NF-κB signal pathways were detected by western blot. The results showed that CPT activated AKT/Nrf2/HO-1 and inhibited NF-κB pathways (**Figures 3A–E**). Then we detected the mRNA levels of M1 markers (IL-6, TNF-α, iNOS, and COX-2) and M2 markers (Ym-1, CD206, and Arg-1) by RT-PCR. Results showed that CPT inhibited the mRNA expression of M1 markers and promoted mRNA expression of M2 markers (**Figures 3F–L**). These results illustrated that CPT treatment inhibits inflammatory response and regulates microglia polarization *via* activating AKT/Nrf2/HO-1 and inhibiting NF-κB pathways in LPS- injected PD mouse model.

### CPT Inhibits the Inflammatory Response in LPS-Exposed BV-2 Cells

**Figure 4:**
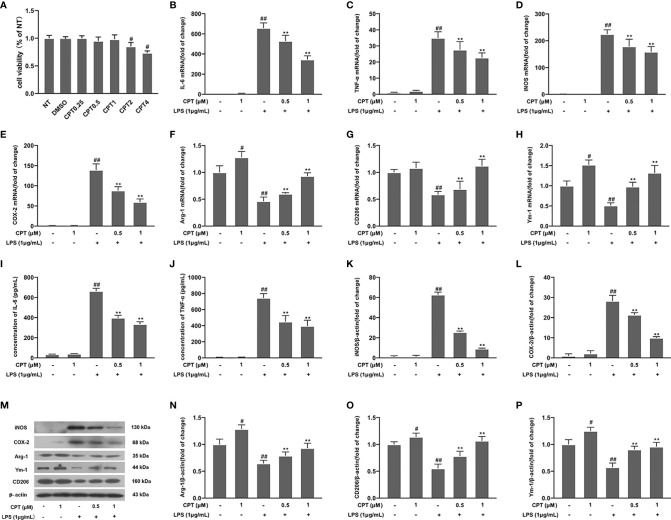
CPT attenuates the inflammatory response in LPS-exposed BV-2 cells. **(A)** The viability of BV-2 cells was measured using CCK-8 assay. The cells were pretreated with CPT (0.5 μM and 1 μM) for 1 h and then treated with LPS (1 μg/mL) for 12 h (mRNA) and 24 h (protein). **(B-H)** The mRNA levels of M1 microglia markers (IL-6, TNF-α, iNOS, and COX-2) and M2 microglia markers (Ym-1, CD206, and Arg-1) were detected by RT-PCR. **(I–P)** The protein levels of M1 microglia markers and M2 microglia markers were measured using ELISA (IL-6 and TNF-α) and western blot (COX-2, iNOS, Arg-1, CD206, and Ym-1). Results are shown as means ± SD (n= 5). *^#^p < 0.5* and *^##^p < 0.01* vs. the no-treatment (NT) group; **p< 0.01 vs. the LPS-exposed group.

To further elucidate the role of CPT on neuroinflammation, we studied the anti-inflammatory effects of CPT on BV-2 cells. First of all, we studied the potential cytotoxic effect of CPT on BV-2 cells. Results showed that CPT for 0-1μM did not affect the viability of BV-2 (**Figure 4A**). Next, we examined the effect of CPT treatment on MI and M2 microglia markers release. The cells were pretreated with CPT for 1 h and stimulated with LPS for 12 h (mRNA) or 24 h (protein) (23). Then the mRNA and protein levels of M1 and M2 markers were tested by RT-PCR, ELISA, and western blot techniques. Results showed that CPT treatment inhibited the expression of MI markers [IL-6 (**Figures 4B, I**), TNF-α (**Figures 4C, J**), iNOS (**Figures 4D, K, M**), and COX-2 (**Figures 4E, L, M**)] and promoted the expression of M2 markers [Arg-1 (**Figures 4F, M, N**), Ym-1 (**Figures 4G, M, P**), and CD206 [**Figures 4H, M, O**)]. These results illustrated that CPT inhibited the inflammatory response in LPS-exposed BV-2 cells.

### CPT Inhibits Activation of NF-κB Pathway in LPS-Exposed BV-2 Cells

**Figure 5:**
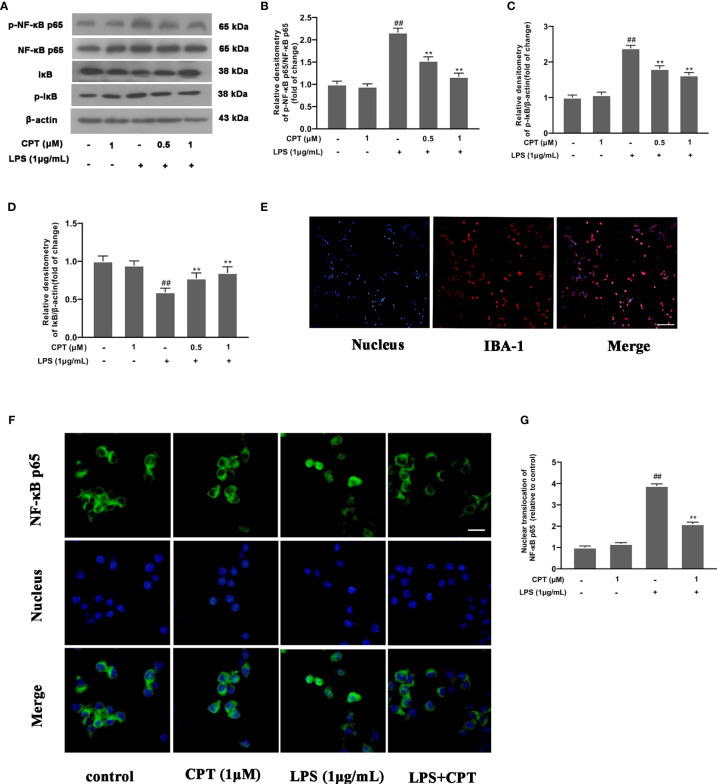
CPT inhibits activation of NF-κB in LPS-exposed BV-2 cells. BV-2 cells were pretreated with CPT (0.5 μM and 1 μM) for 1 h and stimulated with LPS (1 μg/mL) for another 1 h. **(A)**The protein levels of IκB, NF-κB p65, and their phosphorylation were detected by western blot. **(B)** The levels of phos-NF-κB p65 were analyzed relative to NF-κB p65. **(C, D)** The levels of IκB and phos-IκB were analyzed relative to β-actin. **(E)** Microglia were labeled with IBA-1 by immunofluorescence staining (the scale bar represents 100 μm). **(F, G)** The nuclear translocation level of NF-κB p65 was examined by immunofluorescence staining (the scale bar represents 20 μm). Results are shown as means ± SD (n= 5). *^##^p < 0.01* vs. the untreated group (NT); **p < 0.01 vs. the LPS-exposed group.

NF-κB pathway, a key pathway of inflammation, affects the production of many pro-inflammatory mediators. To investigate the mechanism by which CPT regulates the polarization of M1 and M2 microglia, we detected the effect of CPT on the activation of NF-κB pathway. BV-2 cells were pretreated with CPT for 1 h and stimulated with LPS for a further 1 h (24). Then phosphorylation of IκB and NF-κB p65 and degradation of IκB were detected using western blot. After that, we marked microglia with IBA-1 (**Figure 5E**) and examined the nuclear positioning of NF-κB p65 by immunofluorescence. Results showed that CPT inhibited phosphorylation of NF-κB p65 (**Figures 5A, B**) and IκB (**Figures 5A, C**) and degradation of IκB (**Figures 5A, D**) and nuclear translocation of NF-κB p65 (**Figures 5F, G**). These results illustrated that CPT inhibits activation of NF-κB in LPS-exposed BV-2 cells.

### CPT Promotes Phosphorylation of AKT, Activation of Nrf2 and Up-Regulates the Expression of HO-1 in BV-2 Cells

**Figure 6:**
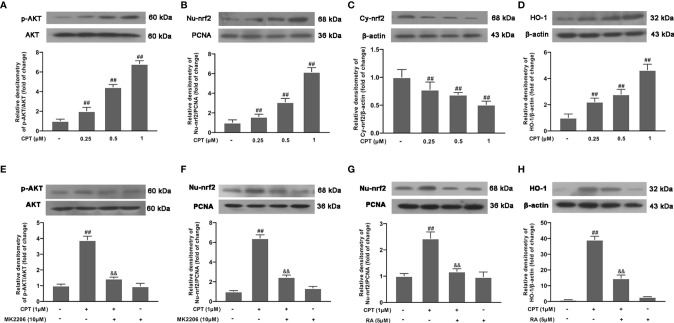
CPT promotes phosphorylation of AKT, activation of Nrf2, and up-regulates the expression of HO-1. **(A–D)** After BV-2 cells were treated with CPT (0, 0.25, 0.5, and 1μM) for 3 h, the phosphorylation of AKT and the protein levels of nuclear-nrf2, Cytoplasm-nrf2, and HO-1 were examined using western blot. **(E, F)** After the cells were treated with MK2206 (an AKT inhibitor, 10μM) for 4 h and CPT (1 μM) for another 3 h, the protein levels of phos-AKT and nuclear-nrf2 were determined by western blot. **(G, H)** After BV-2 cells were treated with RA (a nrf2 inhibitor, 5μM) for 4 h and CPT (1μM) for another 3 h, the protein levels of nuclear-nrf2 and HO-1 were determined by western blot. Results are shown as means ± SD (n= 5). *^##^p < 0.01* vs. the no-treatment (NT)group; *^&&^p < 0.01* vs. the CPT-treated group.

To further clarify the mechanism of CPT anti-inflammation, we studied the effect of CPT on AKT, Nrf2, and HO-1 inflammation pathways. After the cells were treated with CPT (0, 0.25, 0.5, and 1 μM) for 3 h, the protein levels of phos-AKT, HO-1, nuclear-nrf2, and Cytoplasm-nrf2 were detected by western blot. Results showed that CPT treatment promoted phosphorylation of AKT, activation of Nrf2, and up-regulated protein levels of HO-1 (**Figures 6A–D**). Then we pretreated cells with MK2206 (an AKT inhibitor, 10 μM) for 4 h and studied the effect of CPT on activation of Nrf2 using western blot. Results showed that MK2206 inhibited the promotion effect of CPT on the protein level of nuclear-nrf2 (**Figures 6E–F**). We pretreated cells with RA (a nrf2 inhibitor, 5 μM) for 4 h and studied the effect of CPT on protein levels of HO-1. Inhibitor treatment time reference reagents used in Shang and Huang et al (24, 25). Results showed that RA inhibited the effect of CPT on up-regulation of HO-1 (**Figures 6G, H**). These results illustrated that CPT promotes AKT/Nrf2/HO-1 signaling pathways in BV-2 cells.

### CPT Regulates Microglial Polarization *via* Activating AKT/Nrf2/HO-1 and Inhibiting NF-κB Pathways

**Figure 7:**
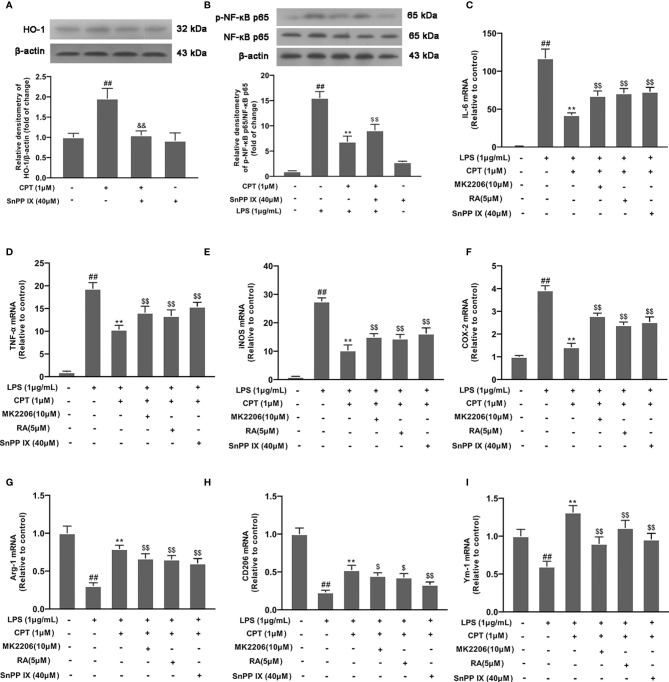
CPT regulates microglial polarization *via* activating AKT/Nrf2/HO-1 and inhibiting NF-κB pathways. **(A)** After the cells were treated with SnPP IX (a HO-1 inhibitor, 40 μM) for 3 h and CPT (1μM) for another 3 h, the protein level of HO-1 was examined by western blot. **(B)** After SnPP IX treatment, the effect of CPT on NF-κB activation was detected by western blot. **(C-I)** The cells were treated with different inhibitors (MK2206 for 4 h, RA for 4 h or SnPP IX for 3 h) and CPT (1μM) for 1 h, then stimulated with LPS for 12 h. The mRNA levels of M1 markers (IL-6, COX-2, iNOS, and TNF-α) and M2 markers (CD206, Ym-1, and Arg-1) were examined using RT-PCR. Results are shown as means ± SD (n= 5).*^##^p < 0.01* vs. the no-treatment (NT) group; **p< 0.01 vs. the LPS-exposed group; *^$^p< 0.05* and *^$$^p < 0.01* vs. the LPS+CPT-treated group.

We pretreated cells with SnPP IX (a HO-1 inhibitor, 40 μM) for 3 h and detected HO-1 expression by Western blot. Results showed that SnPP IX successfully inhibited the expression of HO-1(**Figure 7A**). Then we studied the effect of CPT on NF-κB pathway activation. Results showed that SnPP IX can reverse the effect of CPT on inhibition of NF-κB pathway activation (**Figure 7B**). Then we pretreated BV-2 cells with different inhibitors (MK2206 for 4 h, RA for 4 h, or SnPP IX for 3 h) and studied the effect of CPT on microglial polarization. Results showed that the inhibitors (MK2206, RA, and SnPP IX) can reverse the regulatory effect of CPT on the release of MI markers (IL-6, TNF-α, COX-2, and iNOS) (**Figures 7C–F**) and M2 markers (CD206, Ym-1, and Arg-1) (**Figures 7G–I**). The above results illustrated that CPT regulates microglial polarization *via* activating AKT/Nrf2/HO-1 and inhibiting NF-κB pathways.

### CPT Exerts a Neuroprotective Effect in SHSY5Y and MN9D Cells by Regulating the Polarization of Microglia

**Figure 8:**
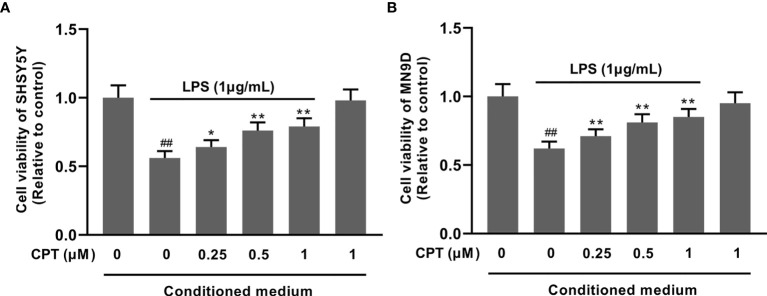
CPT exerts a neuroprotective effect in SHSY5Y and MN9D cells by regulating the polarization of microglia. The SHSY5Y and MN9D cells were cultured with the conditioned medium for 18 h. The viability of the SHSY5Y **(A)** and MN9D **(B)** cells was measured using CCK-8. Results are shown as means ± SD (n= 5). *^##^p < 0.01* vs. the no-treatment (NT) group; **p < 0.01 vs. the LPS-exposed group. **p < 0.01 vs. the LPS-exposed group corrected to *p < 0.05 and **p < 0.01 vs. the LPS-exposed group.

To confirm the effect of CPT on neurons, we studied the effect of CPT-treated BV2 cell supernatant on neuron viability. Firstly, BV-2 cells were preprocessed with CPT (0.25 μM, 0.5 μM and 1 μM) for 1 h and stimulated with LPS (1 μg/mL) for another 3 h. Then we changed the medium and cultured the cells for 18 h under the new medium environment. After that, the supernatant was collected and the conditioned medium was prepared with the supernatant and complete medium in a 1:1 ratio. Then the SHSY5Y and MN9D cells were cultured with the conditioned medium for 18 h. After that, the viability of the SHSY5Y and MN9D cells was measured using CCK-8. The results showed that CPT exerts a neuroprotective effect in SHSY5Y (**Figure 8A**) and MN9D (**Figure 8B**) cells by regulating the polarization of microglia.

## Discussion

PD, the second most common neurodegenerative disease, seriously affects the physical and mental health of elderly people. Clinically, PD patients have severe motor impairments and massive loss of dopaminergic neurons in the SN of the midbrain (26). The etiology of PD is still unclear, and accumulated evidence demonstrates that neuroinflammation plays an important role in the occurrence and development of PD. When neuroinflammation occurs, the immune cells, mainly microglia, are over-activated to release pro-inflammatory mediators, resulting in the degeneration of peripheral neurons (27–29). Therefore, inhibition of neuroinflammation is also considered to be a target for the treatment of PD. LPS, a component of the cell wall of Gram-negative bacteria, can induce inflammation response. Studies have demonstrated that the injection of LPS into the SN of rats can induce PD symptoms (30, 31). In our study, we found that mice injected with LPS in the SN had more PD symptoms, such as weight loss and motor dysfunction, compared with control mice. Further research found that dopaminergic neurons in the SN decreased in LPS-injected mice. Our research also found that CPT could relieve PD symptoms and improve the damage of dopaminergic neurons of LPS-injected mice. The results suggested that CPT has a neuroprotective effect.

Microglia, immune cells in the CNS, are the main participants of the neuro-inflammatory response. Studies have reported that there are numerous microglia activated abnormally in the SN of PD patients and PD model animals (32, 33). Activated microglia were classified as M1-like (showing pro-inflammatory signals and neurotoxicity) or M2-like (involved in remissions of inflammation) based on the presence of specific cell surface molecules and expression of specific cytokines. BV-2 cells, microglial cell lines, have been widely used to study the function of microglia due to the similarity to microglia (34, 35). To further study the neuroprotective effect of CPT and its mechanism, we studied the effect of CPT on microglia inflammation using BV-2 cells. In the experiment, we found that CPT treatment can inhibit the release of pro-inflammatory mediators (IL-6, TNF-α, iNOS, and COX-2) and promote the release of anti-inflammatory mediators (Arg-1, CD206, and Ym-1). We also found that CPT exhibited this effect in LPS-injected mice. The results suggested that CPT inhibits the pro-inflammatory microglia and promotes the anti-inflammatory microglia, thereby exerting an anti-inflammatory effect.

NF-κB, a classic inflammatory pathway, is involved in cellular inflammation and many nervous system diseases. Studies have reported that NF-κB can regulate the transcription of various pro-inflammatory mediators and is considered to be a target for inflammatory diseases (36, 37). There are also reports that NF-κB pathway is closely related to the polarization of immune cells (38). Under normal conditions, NF-κB p65 is present in the cytoplasm and binds to IκB subunit. Once activated, IκB subunit degrades and NF-κB p65 undergoes nuclear translocation and phosphorylation (39). In our study, we found that CPT can inhibit the degradation of IκB and nuclear translocation of NF-κB p65 in LPS-exposed BV-2 cells. The results suggested that CPT inhibits microglia inflammation by repressing the activation of the NF-κB pathway.

AKT, or PI3K-AKT pathway, is involved in fundamental cellular processes including protein synthesis, proliferation, and survival. AKT signaling has been implicated in various inflammation responses (40, 41). Nrf2 is a key transcription factor regulating oxidative stress. Studies found that activated-nrf2 enter into the nucleus and regulates transcription of many inflammatory factors. HO-1 is a key anti-inflammatory protein downstream of Nrf2 (42, 43). In our study, we found that CPT treatment can activate the AKT pathway, promote activation of Nrf2, and up-regulate the expression of HO-1. To further study the anti-inflammatory effect of CPT on microglia, we treated BV-2 cells with MK2206 (an AKT inhibitor), RA (a Nrf2 inhibitor), or SnPP IX (a HO-1 inhibitor). The results showed that, after blocking the AKT pathway with MK2206, the effect of CPT on Nrf2 nuclear transcription was suppressed. This indicates that CPT promotes activation of Nrf2 through activating AKT in microglia. At the same time, RA and SnPP IX treatment also demonstrated the regulatory effect of CPT on inflammatory pathways. In addition, we also explored the effect of CPT on the release of pro-inflammatory and anti-inflammatory mediators in microglia after BV2 cells treated with MK2206, RA, or SnPP IX. The results showed that MK2206, RA, or SnPP IX treatment reversed the regulatory effect of CPT on pro-inflammatory and anti-inflammatory mediators to a certain extent. The results prompted that CPT regulates the polarization of microglia through activating AKT/Nrf2/HO-1 and inhibiting NF-κB pathways. The experiments *in vivo* also confirmed this. Studies have shown that CPT is an inhibitor of expression of Topoisomerase 1. However, no association between Topoisomerase 1 and AKT pathways has been reported. This may be an idea to further study the anti-inflammatory effect of CPT.

To explore the neuroprotective effect of CPT, we pretreated BV-2 cells with CPT and stimulated them with LPS to induce microglial activation. Then we changed the fresh medium for 18 h and obtained conditioned medium. Next we studied the effect of conditioned medium on the viability of SHSY5Y and MN9D neuronal cell lines. The results showed that CPT could inhibit microglial activation-mediated neurotoxicity and protect neurons.

The phenotype of activated microglia has always been controversial. Recently, microglial transcriptome analysis in AD patients and AD models has shown that inhibition of TGF-β signaling is associated with neurodegeneration, leading to a phenotype known as “MGnD” (microglial neurodegenerative phenotype). In further phenotypic regulation studies, it was found that the phenotypic switch of microglia is regulated by the activation of TREM2-apo E signaling by phosphatidylserine exposure to apoptotic neurons. Not all microglia express the triggering receptor expressing myeloid cell 2 (TREM2). Therefore, according to the expression of TREM2, the activated microglia have been given a new name: disease-associated phenotype (DAM) (44, 45). However, it remains controversial whether MGnD or DAM microglia are beneficial or harmful in neurodegenerative diseases. The regulation of microglia-related phenotypes has become a research direction of targeting microglia cells in the treatment of neurodegenerative diseases. In our experiment, we found that CPT improved the symptoms of PD mice. The mechanism study found that CPT regulated the release of pro-inflammatory and anti-inflammatory mediators, suggesting that it inhibited the pro-inflammatory microglia phenotype and promoted the anti-inflammatory microglia phenotype. However, it is not known whether CPT regulates MGnD or DAM microglia-related genes. This provides a new idea for further study on the anti-inflammatory effect of CPT.

## Conclusions

**Figure 9:**
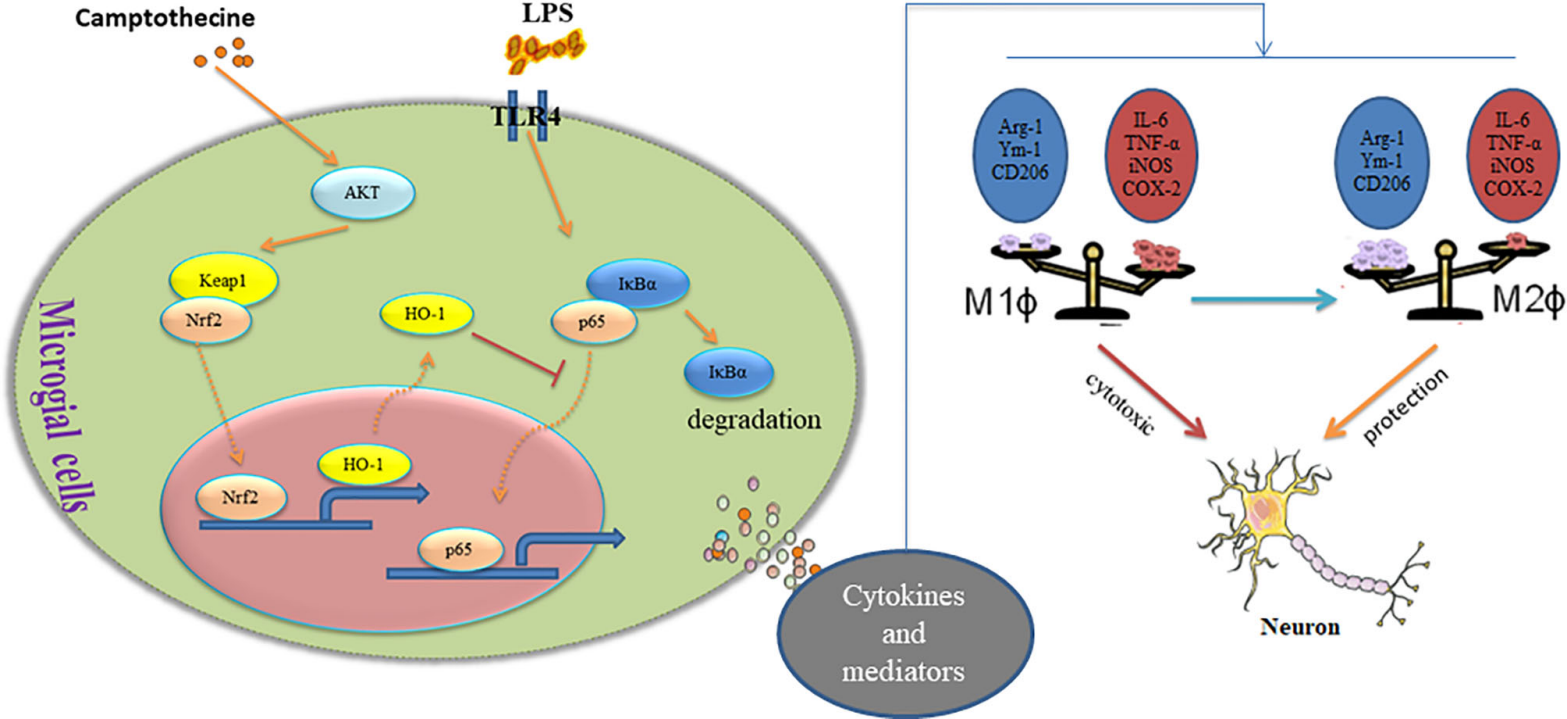
Camptothecin regulates microglia polarization and exerts neuroprotective effects via the AKT/Nrf2/HO-1-NF-κB signal axis.

In conclusion, our study found for the first time that CPT inhibits microglial M1 polarization and promotes M2 polarization through the AKT/Nrf2/HO-1-NF-κB signal axis, thereby inhibiting neuroinflammation and exerting the effect of neuroprotection *in vivo* and *in vitro* (**Figure 9**). This experiment would provide new ideas for the treatment of inflammation-mediated PD. Combining its extensive pharmacological effects and easily available properties, CPT is expected to be further developed and studied. Future research will also focus more on the potential therapeutic role of CPT in inflammatory diseases and assess the possibility of being developed as anti-inflammatory drugs.

## Author’s Note

This manuscript has been released as a pre-print at ResearchGate [10.21203/rs.3.rs-46120/v1] (46).

## Data Availability Statement

The datasets presented in this study can be found in online repositories. The names of the repository/repositories and accession number(s) can be found in the article/**Supplementary Material**.

## Ethics Statement

The animal study was reviewed and approved by Institutional Animal Care and Use Committee of Jilin University (Changchun, China) (Permit Number: 2015047).

## Author Contributions

DH and SF accomplished most of the experiments, analyzed the results, and wrote the manuscript. DL designed this study. AZ and YS took part in various aspects of the study and read and revised first draft. XG, YZ, BH, and JD also participated in the research. All authors contributed to the article and approved the submitted version

## Funding

This work was funded by the National Natural Science Foundation of China (project No. 31772547, 31702211) and the Jilin Scientific and Technological Development Program (project No. 20200703011ZP).

## Conflict of Interest

The authors declare that the research was conducted in the absence of any commercial or financial relationships that could be construed as a potential conflict of interest.

## Abbreviations

AKT, protein kinase B; Arg-1, Arginase 1; CD206, Mannose receptor; COX-2, Cyclooxygenase-2; CNS, Central nervous system; HO-1, Hypoeretin-1; iNOS, Induced nitric oxide synthase; IL-6, Interleukin-6; PD, NF-kB, nuclear factor kappa B; Nrf2, nuclear factor erythroid 2-related factor 2; Parkinson’s disease; RA, retinoic acid; SN, Substantia nigra; SnPP IX, tin protoporphyrin-IX; TNF-α, Tumor necrosis factor-α; Ym-1, Chitinase-3-Like-3.
