# Supplementary figures and images for: Camptothecin Regulates Microglia Polarization and Exerts Neuroprotective Effects via Activating AKT/Nrf2/HO-1 and Inhibiting NF-κB Pathways In Vivo and In Vitro
Source: Front Immunol. 2021 Apr 1;12:619761. doi: 10.3389/fimmu.2021.619761 (PMC8047064; doi:10.3389/fimmu.2021.619761)

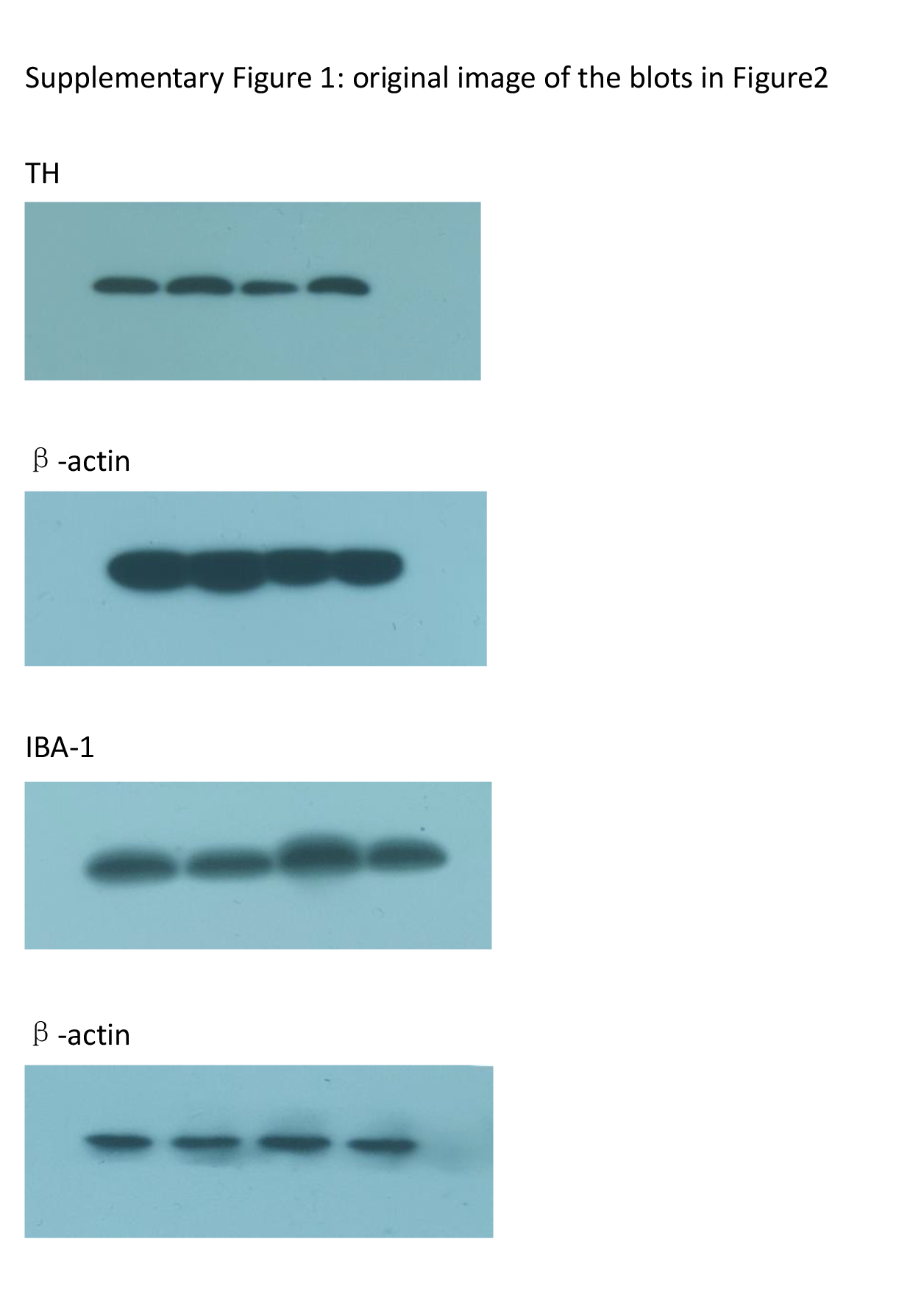

Supplement: Supplementary file 1 [file Image_1.tif]

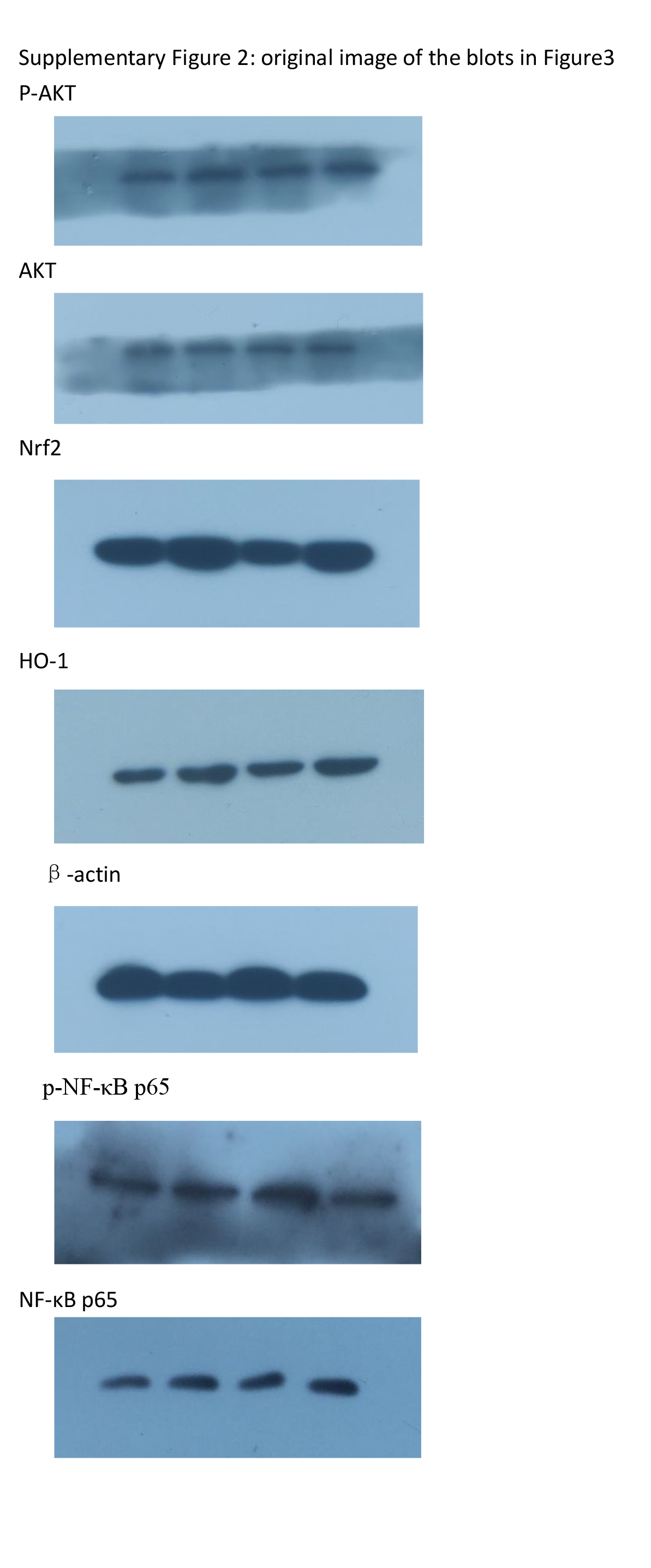

Supplement: Supplementary file 2 [file Image_2.tif]

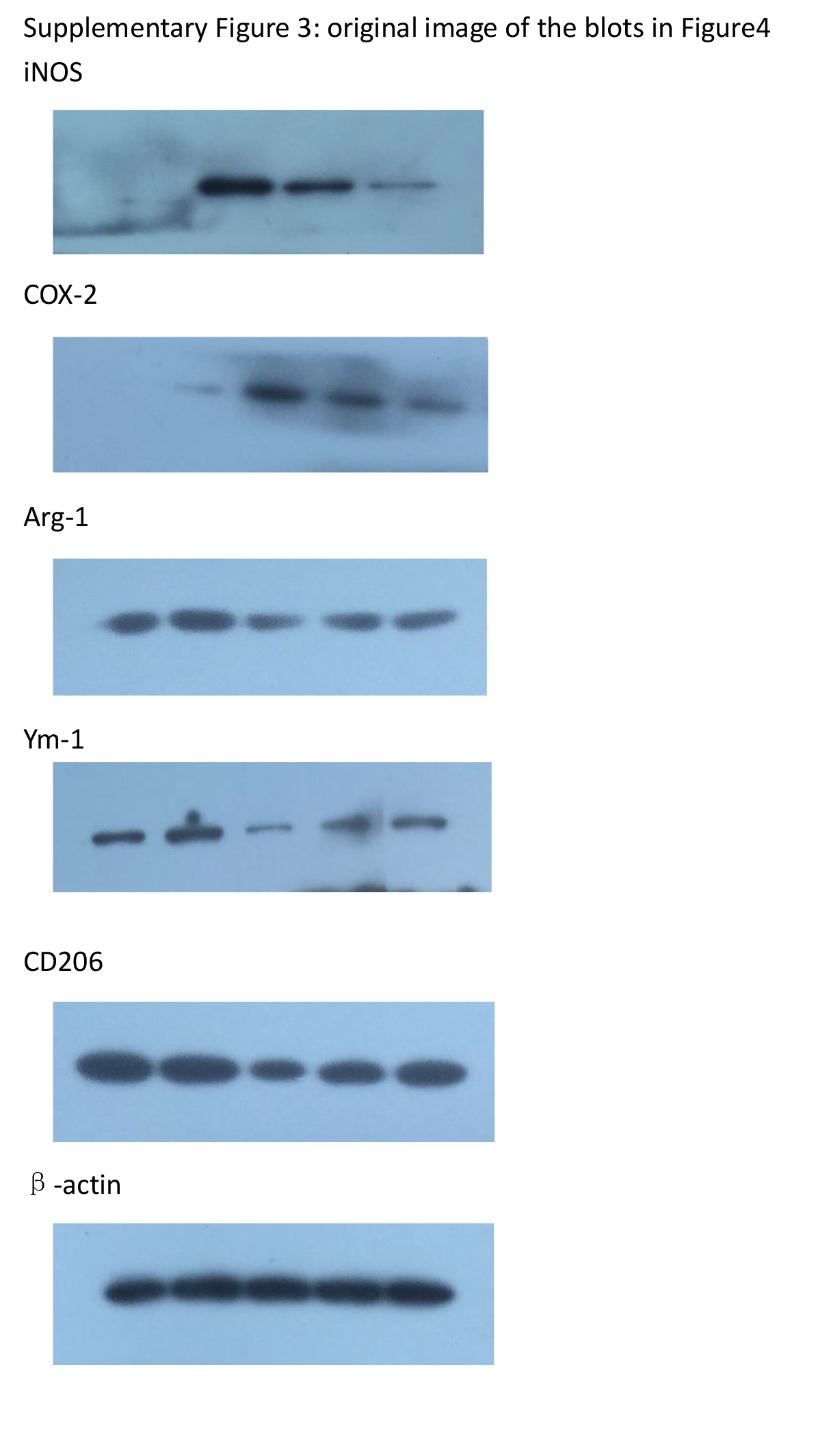

Supplement: Supplementary file 3 [file Image_3.tif]

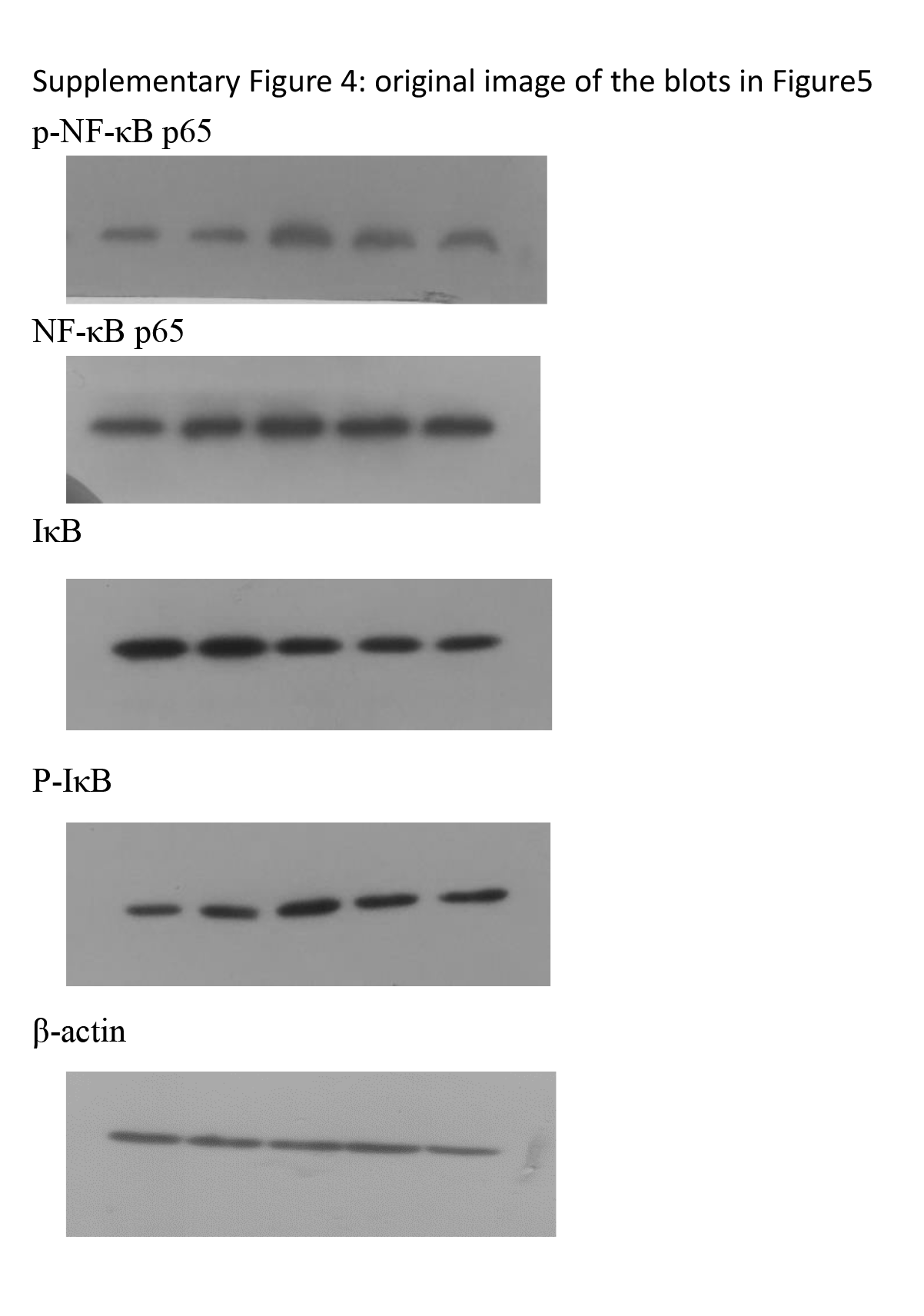

Supplement: Supplementary file 4 [file Image_4.tif]

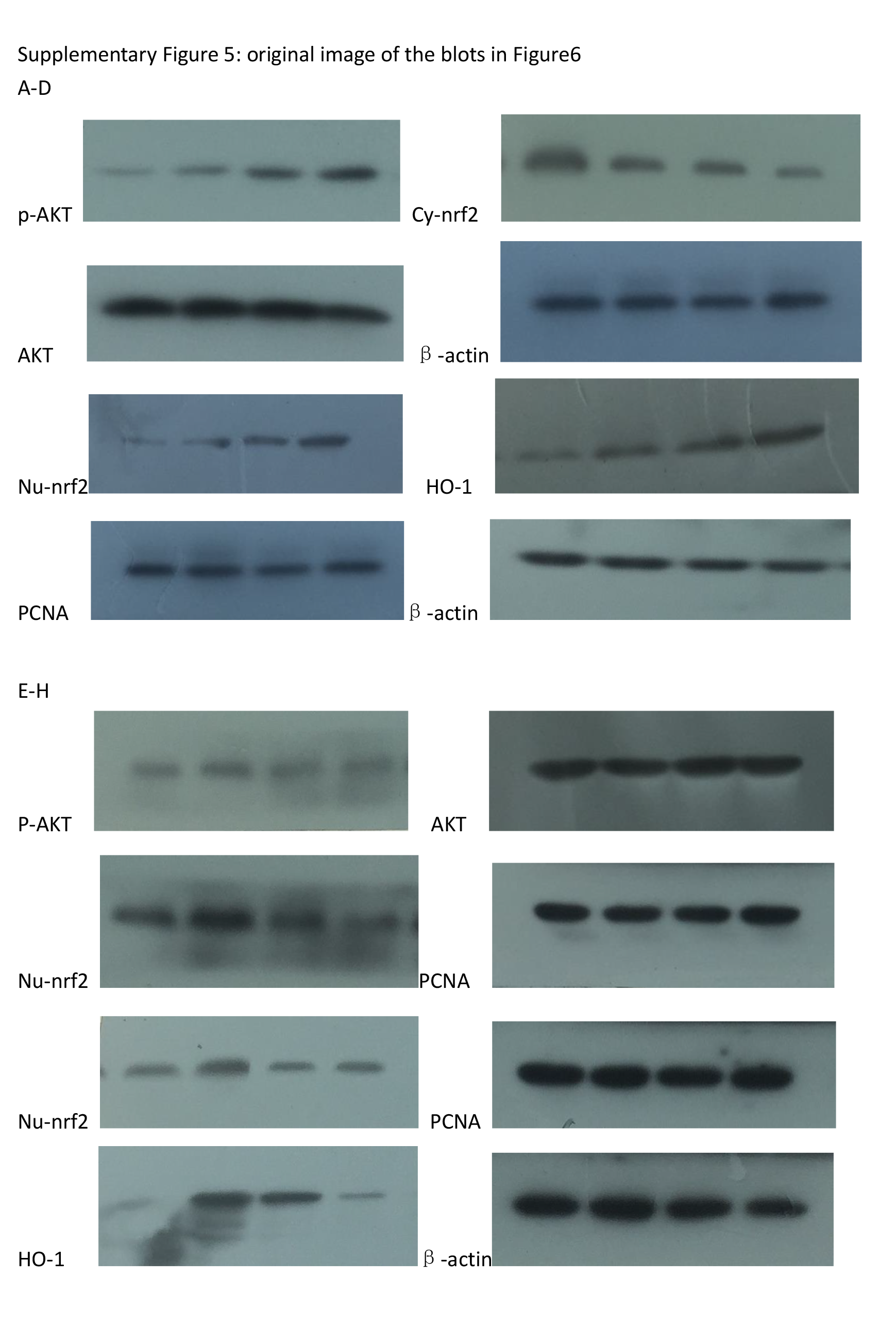

Supplement: Supplementary file 5 [file Image_5.tif]

Figure2

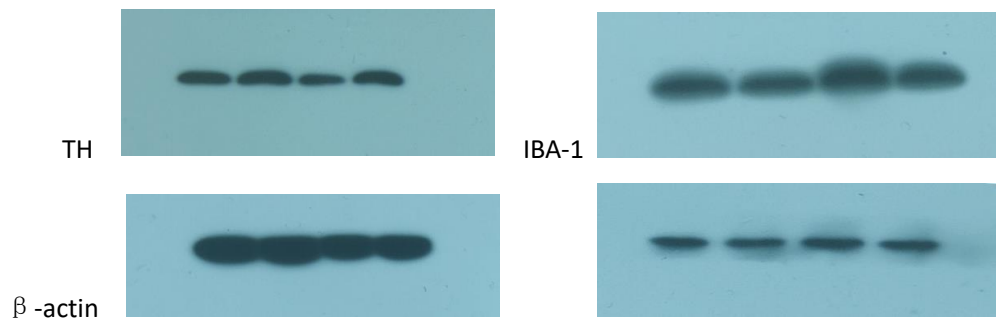

Figure3.

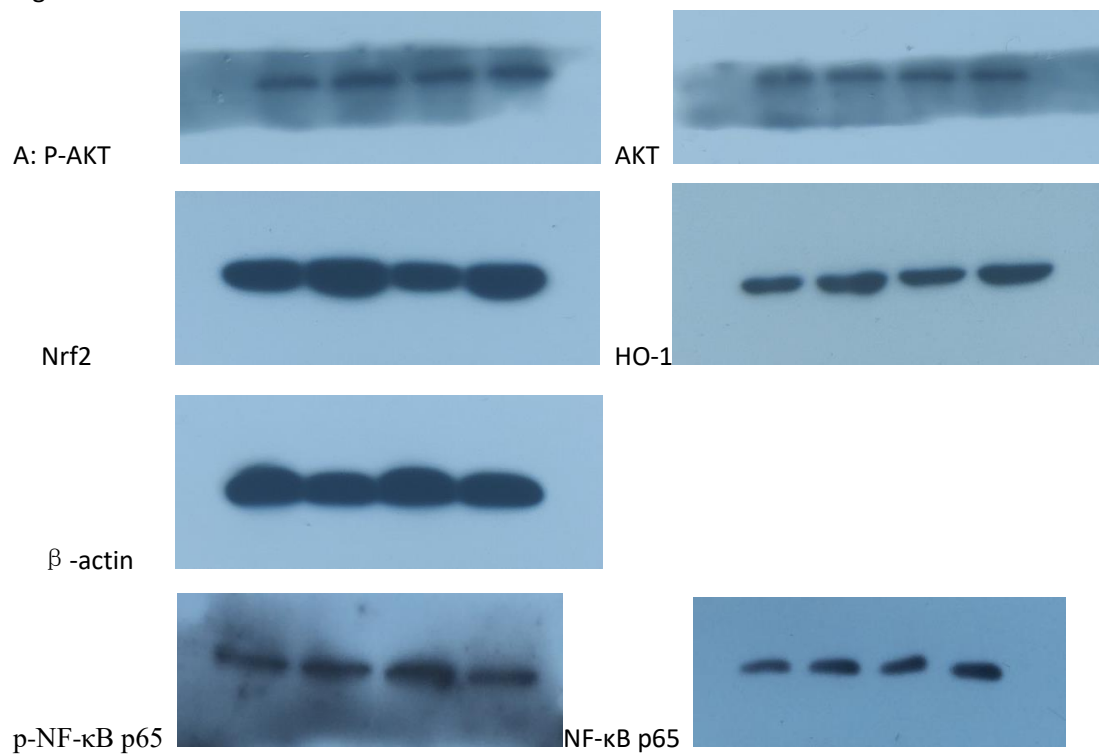

Figure4.

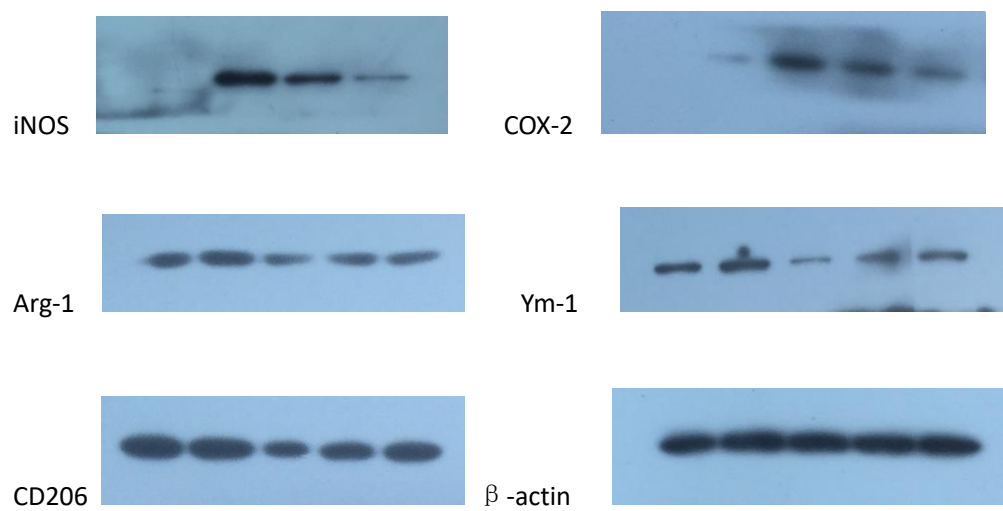

Figure5.

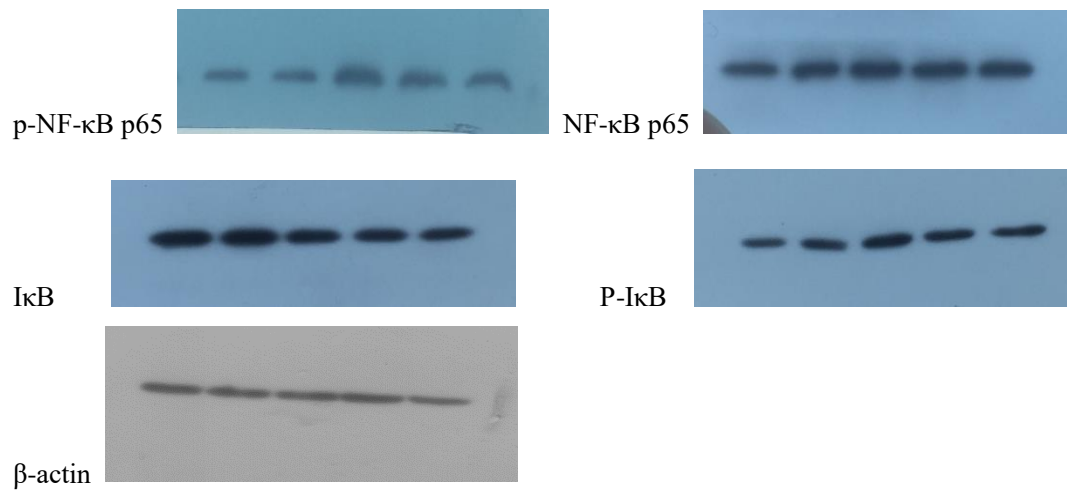

Figure6.

A-D

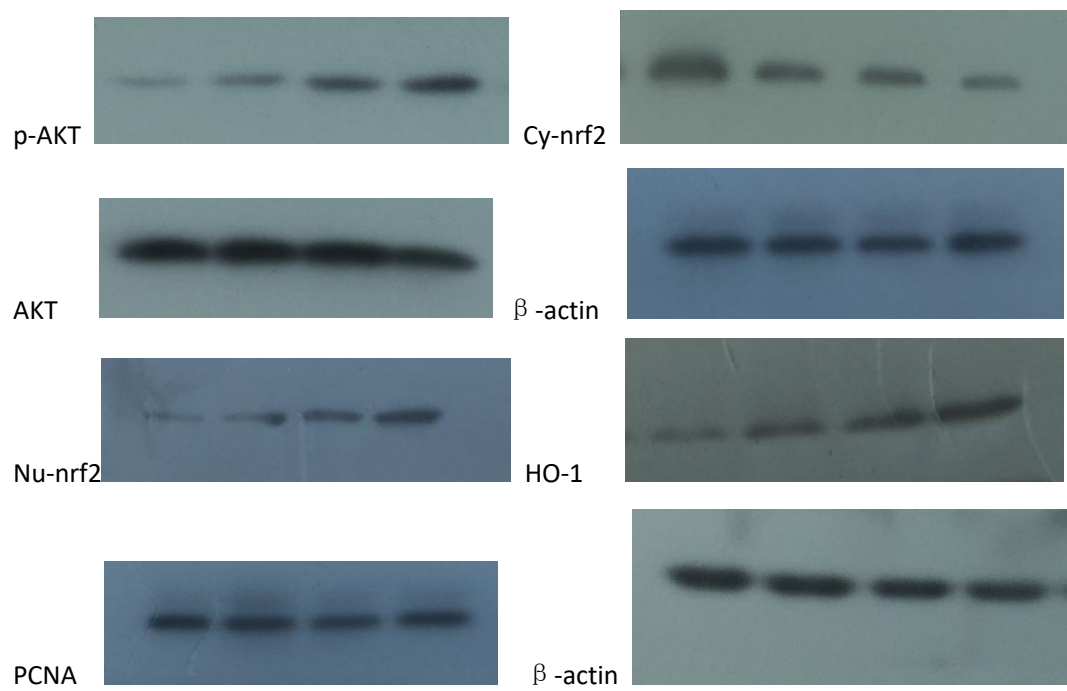

E-H

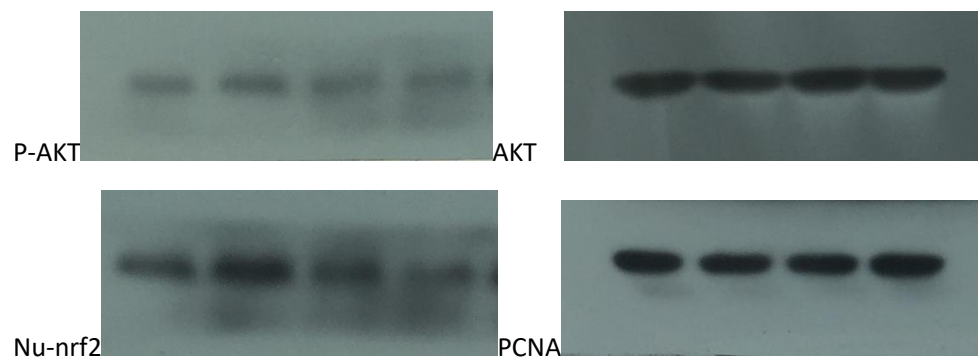

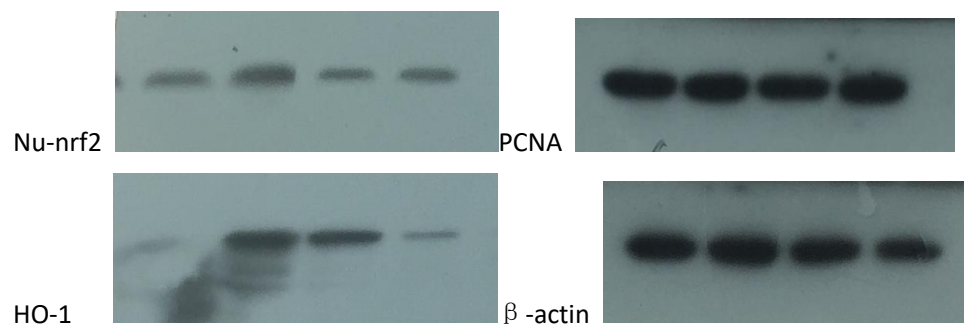

Figure7.

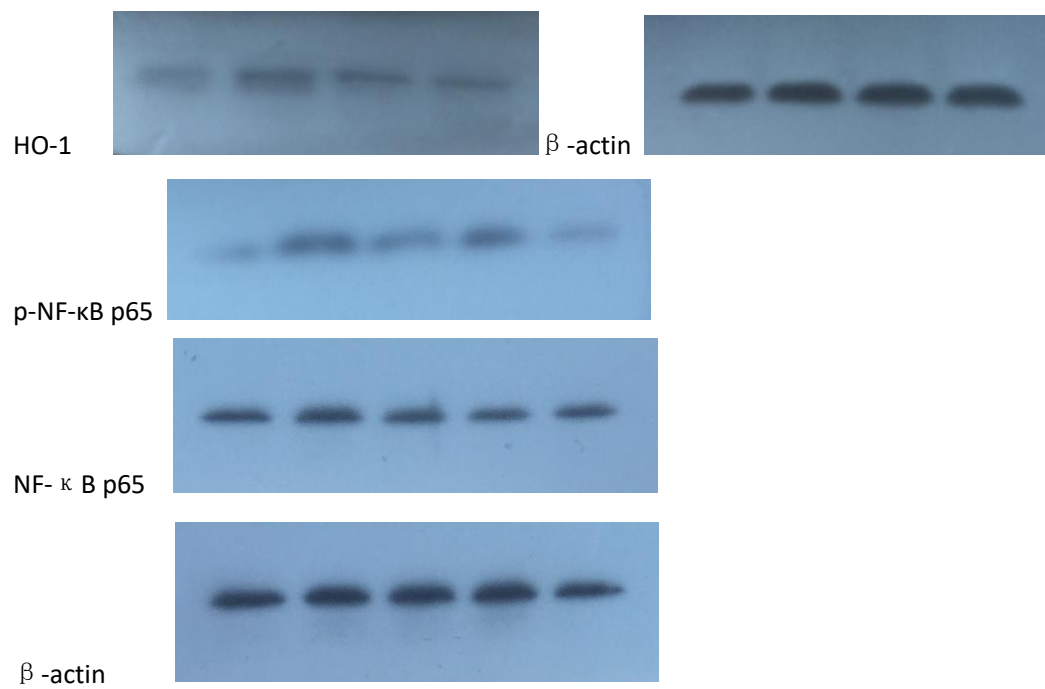

Supplement: Supplementary file 6 [file DataSheet_1.pdf]
